# Antipyrin

**Published:** 1890-09-06

**Authors:** 


### THERAPEUTICS.

### ANTIPYRIN.

In the "Retrospect" for October 5th last we referred to various toxic effects which have been noticed after the use of antipyrin and antefibrin. The principal unpleasant effects which have been noticed after antipyrin are burning in the throat, dizziness, loss of vision, dyspnœa, irregularity of the action of the heart, vomiting, cyanosis, and eruptions on the skin. It must be now generally admitted that the action of antipyrin is somewhat uncertain, and may be dangerous. Death even has occurred after its administration (*Lancet*, 1885). We have therefore on several occasions (notably "Retrospect," October 26th and November 9th) cautioned against the indiscriminate or excessive use of antipyrin. A writer in the *Practitioner*, some time ago, reported the drug as especially useful for children. But children are particularly susceptible to its influence, and we think that more than a grain or a grain and a-half should not be given until experience shows there is no individual idiosyncracy, rendering the child more than ordinarily susceptible. A writer in the *British Medical Journal* stated that antipyrin should be avoided when the kidneys are diseased, and with this we fully agree. It would seem that the conditions under which unfavourable results follow are not yet sufficiently known. In some instances it may probably be due to an inferior preparation. In the same journal there is a communication on the "Risk from Impurities," but, perhaps, in most cases, unfavourable results are due to peculiar constitution or condition of the patient. Antipyrin has been spoken of as very beneficial for dysmenorrhœa, and especially for neuralgic or painful dysmenorrhœa, but several cases have recently been reported of injurious effects resulting to menstruating females from its use. Dr. W. Huchard reports (*Revue Gènèrale de Therapeutique*) that he administered fifteen grains of antipyrin to a woman suffering from violent dysmenorrhœa. As the result of the administration of the drug the menstrual flow was suddenly checked. The patient was seized with violent chill and chattering of the teeth, the face became cyanosed, there were frequent attacks of syncope, the pulse was small and weak, and she complained greatly of headache. The condition was such as to cause great anxiety for nearly an hour, when the effects gradually passed off. Dr. Huchard has observed similar symptoms, although less marked, in two other cases, and he now regards the presence of the catamenial flow as a positive contra-indication to the use of antipyrin.

